# COL11A1 serves as a biomarker for poor prognosis and correlates with immune infiltration in breast cancer

**DOI:** 10.3389/fgene.2022.935860

**Published:** 2022-09-09

**Authors:** Qi Luo, Jinsui Li, Xiaohan Su, Qiao Tan, Fangfang Zhou, Shaoli Xie

**Affiliations:** ^1^ Department of Academician (expert) Workstation, Affiliated Hospital of North Sichuan Medical College, Nanchong, Sichuan, China; ^2^ Biological Targeting Laboratory of Breast Cancer, Affiliated Hospital of North Sichuan Medical College, Nanchong, Sichuan, China; ^3^ Department of Outpatient, Affiliated Hospital of North Sichuan Medical College, Nanchong, Sichuan, China; ^4^ Department of Thyroid and Breast Surgery, Affiliated Hospital of North Sichuan Medical College, Nanchong, Sichuan, China; ^5^ Department of Anatomy, North Sichuan Medical College, Nanchong, Sichuan, China

**Keywords:** COL11A1, breast cancer, immune infiltration, prognosis, bioinformation

## Abstract

Breast cancer is the malignant tumor with the highest incidence rate at present, and its incidence rate ranks first in the female population. COL11A1 is an important component of collagen XI and is considered to play an important role in a variety of connective tissue diseases. Recent studies have shown that COL11A1 is associated with the occurrence and development of many kinds of malignant tumors. However, its prognostic value in breast cancer and its correlation with immune cell infiltration in tumor tissue are not clear. In this paper, we reveal the prognostic value of COL11A1 in breast cancer and its tumor immune-related function through in-depth bioinformatics analysis. The expression of COL11A1 is abnormally upregulated in breast cancer and is significantly related to the poor prognosis of breast cancer. In the analysis of the clinical characteristics of the patients, we found that the expression level of COLL11A1 was closely related to lymph node metastasis, PAM50 (Prediction Analysis of Microarray 50) expression, clinical stage and so on. Gene Ontology (GO) and Kyoto Encyclopedia of Gene and Genome (KEGG) all suggest that COL11A1 is related to tumor immunity. Further study found that the COL11A1 expression was significantly correlated with the degree of immune infiltration and the expression of a variety of immune cell markers in tumor tissue. More importantly, COL11A1 can affect the prognosis of breast cancer patients by participating in the regulation of tumor immune infiltration. Therefore, we believe that COL11A1 is a very potential target for diagnosis and treatment of breast cancer.

## Introduction

Breast cancer is an important cause of cancer-related death among women around the world (Miller et al., 2021; Sung et al., 2021; Siegel et al., 2022). According to the latest global cancer statistics, breast cancer has become the first malignant tumor in the female population in terms of morbidity and mortality (Desantis et al., 2019; Sung et al., 2021). With the progress of modern medical science and technology and the deepening of people’s understanding of breast cancer, the treatment of breast cancer has evolved from the earliest single surgical treatment to a variety of combined treatment (Desantis et al., 2019; Waks and Winer, 2019). Especially since anthracyclines and taxanes are used as the cornerstone of breast cancer chemotherapy, the therapeutic effect of early breast cancer has made great progress. However, the pathogenesis of breast cancer is insidious, and there is a lack of corresponding clinical symptoms and effective diagnostic methods in the early stage. According to statistics, most of the breast cancer diagnosed clinically for the first time are advanced breast cancer (Banham et al., 2019; Franzoi et al., 2019; Sabik et al., 2020). At present, the comprehensive treatment strategies advocated all over the world, which are mainly surgical treatment, supplemented by chemotherapy, endocrine therapy and targeted therapy, have not shown excellent therapeutic effects (Gradishar et al., 2018). Therefore, finding new prognostic factors and treatment targets of breast cancer is an urgent problem to be solved in the process of diagnosis and treatment of breast cancer.

Collagen is a kind of protein family peculiar to animals, accounting for about 20% of the total protein of mammals. Collagen is also one of the main components of human connective tissue. Up to now, people have discovered at least 30 kinds of collagen chain encoding genes, which can form more than 16 kinds of collagen molecules. According to their structures, they can be divided into fibrous collagen, basal membrane collagen, microfibrous collagen, anchored collagen, hexagonal reticular collagen, non-fibrous collagen, transmembrane collagen, etc. By function, collagen could be divided into two groups. The first group is fibroblast collagen, including 6 types of collagen (type I, II, III, XI, etc). And the test constitutes the second group called non-fibroblasts. The α -chain of non-fibroblast collagen contains both the triple helix domain (COL) and the non-triple helix domain (NC), in which the former accounts for about 90% of the non-fibroblast. Collagen can be involved in many biological processes of wound healing. In the early stage of wound formation, collagen interacts with platelets to form hemostatic clots. The collagen in the scab has a natural antibacterial effect, and the aseptic state maintained by collagen provides the most basic microenvironment for wound recovery. The existing studies show that collagen deficiency is closely related to a variety of connective tissue diseases such as Marshall-Stickler Syndrome (Bath et al., 2021; Mladenova et al., 2021) and Ehlers-Danlos syndrome (Micale et al., 2020). In recent years, some collagen proteins have attracted the attention of scholars in the oncology field since they are considered to be involved in the occurrence and development of tumors.

COL11A1 is the Alpha1 chain of type Xi collagen, and its coding gene is located in band 1, region 2 of the short arm of chromosome 1. More and more studies believe that collagen, as the interstitial component of solid tumor, provides a scaffold for tumor cell proliferation, invasion and metastasis (Mandelbaum, 1967; Wu et al., 2021a; Kang et al., 2021). Coincidentally, a number of previous studies have found a significant increase in the expression of collagen XI α 1 chain in primary lesions including ovarian cancer (Wu et al., 2021b), esophageal cancer (Kang et al., 2021), lung cancer (Sun et al., 2021), colon cancer (Galvan et al., 2014) and pancreatic cancer (Wang et al., 2021). In ovarian cancer, PRRX1 enhances tumor progression and resistance to chemotherapy and radiotherapy by activating COL11A1 (Wu et al., 2019; Zhu et al., 2021a; Heiserman et al., 2021), and miR-335 inhibits the invasion and migration of ovarian cancer cells by inhibiting the expression of COL11A1 (Wu et al., 2021a). Circ-0005105 promotes the development of malignant characteristics of pancreatic cancer cells by targeting miR-20a-3p and activating COL11A1 (Ma et al., 2021). In addition, the overexpression of COL11A1 in bladder cancer also showed a significant correlation with poor prognosis (Zhang and Fu, 2021). Therefore, COL11A1 can be used as a risk factor for a variety of human malignant tumors and a marker for predicting poor prognosis. However, there are few studies on COL11A1 in human breast cancer (Gu et al., 2019; Wang et al., 2020a). Especially its invasive development and the relationship with immune infiltration in breast cancer are still in a vacuum.

Given about that COL11A1 is closely related to the occurrence and development, drug resistance and poor prognosis of many kinds of tumors, there is only limited evidence that COL11A1 may also be involved in the pathogenesis of breast cancer. The purpose of this study is to elucidate the relationship between COL11A1 and breast cancer progression and immune infiltration through bioinformatics methods, and to explore its molecular regulation mechanism. In this study, we found that COL11A1 was significantly up-regulated in breast cancer tissues, whether in independent samples or paired samples, and the high expression of COL11A1 was significantly associated with poor prognosis of breast cancer patients. Tumor immune infiltration analysis showed that the expression level of COL11A1 in breast cancer tissue was closely related to the infiltration degree of many kinds of immune cells in tumor tissue. Further immune infiltration related survival analysis suggested that COL11A1 may affect the prognosis of breast cancer patients by affecting the infiltration level of some immune cells. These observations emphasize the important role of COL11A1 in the occurrence and development of breast cancer and suggest that it may be involved in the regulation of immune cell infiltration in breast cancer.

## Materials and methods

### Acquisition and preservation of clinical pathological tissue samples

Breast cancer tissue samples and their paired paracancerous tissue samples in this study were collected from breast cancer patients in the Department of Breast and Thyroid Cancer Surgery, North Sichuan Medical College. All samples retained the primary tumor of breast cancer and its adjacent tissue samples at 3 cm adjacent to the tumor. The samples were divided into two parts, one part was fixed and stored in 4% formalin solution, and the other part was stored in a -80°C refrigerator. The preserved specimens were sent to the pathology room, and the tissue was embedded in paraffin and made into pathological sections with a thickness of 5 μm by a pathological laboratory expert. All samples in this study were obtained with the written permission of the patients themselves, and the entire research process complied with the approval of the Ethics Committee of the Affiliated Hospital of North Sichuan Medical College (Ethics Registration No. 20190707).

### The cancer genome atlas

The Cancer Genome Atlas is a cancer research project established by National Cancer Institute (NCI, National Cancer Institute) and National Human Genome Research Institute (NHGRI, National Human Genome Institute). It provides a large, free cancer research reference database by collecting and collating all kinds of cancer-related genomic data. At present, there are 33 types of cancer, including breast cancer, more than 2 PB data, which is free and available, greatly helping cancer researchers to improve the prevention, diagnosis and treatment of cancer. In this study, the gene expression information of breast cancer samples and adjacent tissue samples were downloaded from the database, as well as the corresponding clinical information (including clinicopathological features). In the analysis of differentially expressed genes, the threshold of adjusting *p* value was set to 0.05 and the absolute value of the threshold of Foldchange was set to 1, and the gene level was set to ALL.

### UALCAN

UALCAN (http://ualcan.path.uab.edu/index.html) is an effective website for online analysis and mining of cancer data, which integrates relevant cancer data based on TCGA database and CPTAC (Clinical Proteomic Tumor Analysis Consortium) (Chen et al., 2019). It can help medical staff to carry out biomarker identification, expression profile analysis, survival analysis and so on. You can also query relevant information in other databases through related links. In a word, it is a fast and efficient website tool for data mining and analysis of TCGA and CPTAC. In this study, UALCAN was used to analyze the protein expression level of COL11A1 in breast cancer and its adjacent tissues.

### Gene expression profiling interactive analysis

GEPIA2 database (Gene Expression Profilling Interactive Analysis2) is a bioinformatics analysis tool that integrates the cancer-related big data of TCGA and the big data of normal tissues in GTEx, and uses bioinformatics technology to solve important problems in cancer biology, and to reveal cancer subtypes, driving genes, alleles, differentially expressed genes or carcinogenic factors, so as to explore new cancer targets and markers (Tang et al., 2019). Generally speaking, GEPIA2 database integrates the current cancer genomic data, which can mine the data more simply and quickly, and dynamically analyze the gene expression profile data. In our study, the data of TCGANormal and GTEx were matched, and log2 (TPM+1) was used as logarithmic scale to investigate the expression of COL11A1 between BRCA and normal breast tissue samples.

### MethSurv

MethSurv (https://biit.cs.ut.ee/methsurv/) is a web tool for survival analysis based on CpG methylation patterns, with 7358 methylation data for 25 different human cancers, using the Cox proportional hazards model to develop an interactive web tool for survival analysis (Modhukur et al., 2018; Wang et al., 2020b; Anuraga et al., 2021; Kao et al., 2021; Zheng et al., 2021). MethSurv enables survival analysis of CpGs located at or near the query gene, and can also provide cluster analysis of the query gene to correlate methylation patterns with clinical features and screen for major biomarkers for each cancer type.

### Tumor immune estimation resource

TIMER (https://cistrome.shinyapps.io/timer/) is a website tool led by Liu Xiaole, a professor of immunoinformatics at Harvard University (Li et al., 2017). The website uses RNA-Seq expression profile to detect the infiltration of immune cells in tumor tissues. TIMER provides the infiltration of a variety of immune cells (B cells, CD4^+^ T cells, CD8^+^ T cells, Neutrphils, Macrophages and Dendritic cells, etc.). In this study, the expression of COL11A1 in a variety of cancers was evaluated by the “diffExp” module. Immediate analysis of the correlation between COL11A1 and immune cell infiltration in BCRA was carried out. Under the “Gene” module, the TCGA database can be used to explore the relationship between the expression of COL11A1 and the level of immune cell infiltration (B cells, CD8+T cells, CD4+T cells, neutrophils, macrophages and dendritic cells). As for the “correlation” module, timer was used to study the relationship between the expression of COL11A1 and different gene marker sets of immune cells. The correlation between COL11A1 expression and immune infiltration was evaluated by purity correlation, Spearman correlation.

### Kaplan-meier plotter database analysis

Based on gene chips and RNA-seq data from public databases such as GEO, EGA and TCGA, the Kaplan-meierPlotter database was constructed (Lanczky and Gyorffy, 2021). And it evaluated the impact of 54,675 genes on survival in 21 cancers, including breast cancer (6234 cases), ovarian cancer (2190 cases), lung cancer (3,452 cases) and gastric cancer (1,440 cases). The Kaplan-meier Plotter database integrates gene expression information and clinical prognostic value for Meta-analysis and to research, discover and verify survival-related molecular markers. In this study, the patient samples were divided into two groups to find the best segmentation site, and the overall survival time (OS), risk ratio (HRs), 95% confidence interval (95%CI) and logarithmic *p* value were analyzed.

### Analysis of COL11A1-interacting genes and proteins

The COL11A1 interaction network was constructed using the GeneMANIA database (http://www.genemania.org) (Warde-Farley et al., 2010). The protein-protein interaction network of COL11A1 was constructed using the STRING online database (https://string-db.org/).

### Immunocytochemistry

Immunohistochemical staining was performed by pathologists. The breast cancer tissue embedded in paraffin blocks was cut into 5 μm sections and dewaxed with gradient alcohol. After the sections were permeated in 0.4%TritonX-100 for 30 min, the antigens were blocked with goat serum for 2 h. After discarding the sealing solution, the slices were incubated with diluted Anti-COL11A1 antibody (ab64883, 1:400, Abcam) overnight at 4°C, and the second antibody (SP-9000, ZSGB-BIO, China) was incubated at room temperature for 1 h the next day. After the second antibody was removed, it was incubated with horseradish peroxidase labeled streptavidin (ZLI9017, ZSGB-BIO, China) for 10 min and the nucleus was re-stained in hematoxylin solution. The staining results were interpreted by two experienced pathologists, whose scores were based on the scoring system proposed by the famous pathologist Friedrichs (Friedrichs et al., 1993).

### Immunofluorescence staining

Tumor tissues were fixed with 4% paraformaldehyde for 12 h at 4°C and then dehydrated in 30% sucrose solution. Tissues were then frozen in OCT (Sakura, Torrance, CA) and sectioned to 5 μm using a cryostat (Leica, Germany). Sections were washed 3 times with phosphate buffered saline (PBS) to remove OCT, and M2-TAMs were detected by immunohistochemical staining with Alexa Fluor 549 anti-human CD206 (BioLegend) or Alexa Fluor 488 anti-human 163 (BioLegend). All sections were imaged with an LSM 710 confocal laser scanning microscope (Zeiss, Germany). Data were analyzed using Image-Pro Plus software.

### Quantitative real-time PCR

Briefly, TRIzol reagent (Thermo Fisher) was used to extract total RNA from tissues, and the RevertAid First Strand cDNA Synthesis Kit was used to generate cDNA by reverse transcription according to the experimental method provided in the kit. The obtained cDNA was subjected to RT-qPCR in the Roche 96 system. In this study, GAPDH was selected as the internal reference, and the primers used in the experiment are shown in Supplementary Table S1. The relative expressions of target genes were calculated by the comparative CT method (2^−ΔΔCT^).

### Statistical analysis

The gene expression data were downloaded from TCGA database, and the differential expression was analyzed by R software package DESeq2 [1.26.0] (Love et al., 2014). The results were displayed by *p* value, foldchange and ranking. R package clusterProfiler package [version 3.14.3] (Wu et al., 2021c) (for enrichment analysis); org. Hs.eg.db package [version 3.10.0] was used for ID conversion, and the species was set to *Homo sapiens*. The results of Kaplan-Meier diagram, PrognoScan and GEPIA2 were displayed by HR and P or COX *p* values of logarithmic rank test. The correlation of gene expression was evaluated by Spearman correlation coefficien. The R software package pheatmap and Spearman’s test were used to generate the heat map of COL11A1 co-expression related genes. *p* < 0.05 is considered to be statistically significant.

## Results

### COL11A1 expression is up-regulated in breast cancer patients

**FIGURE 1:**
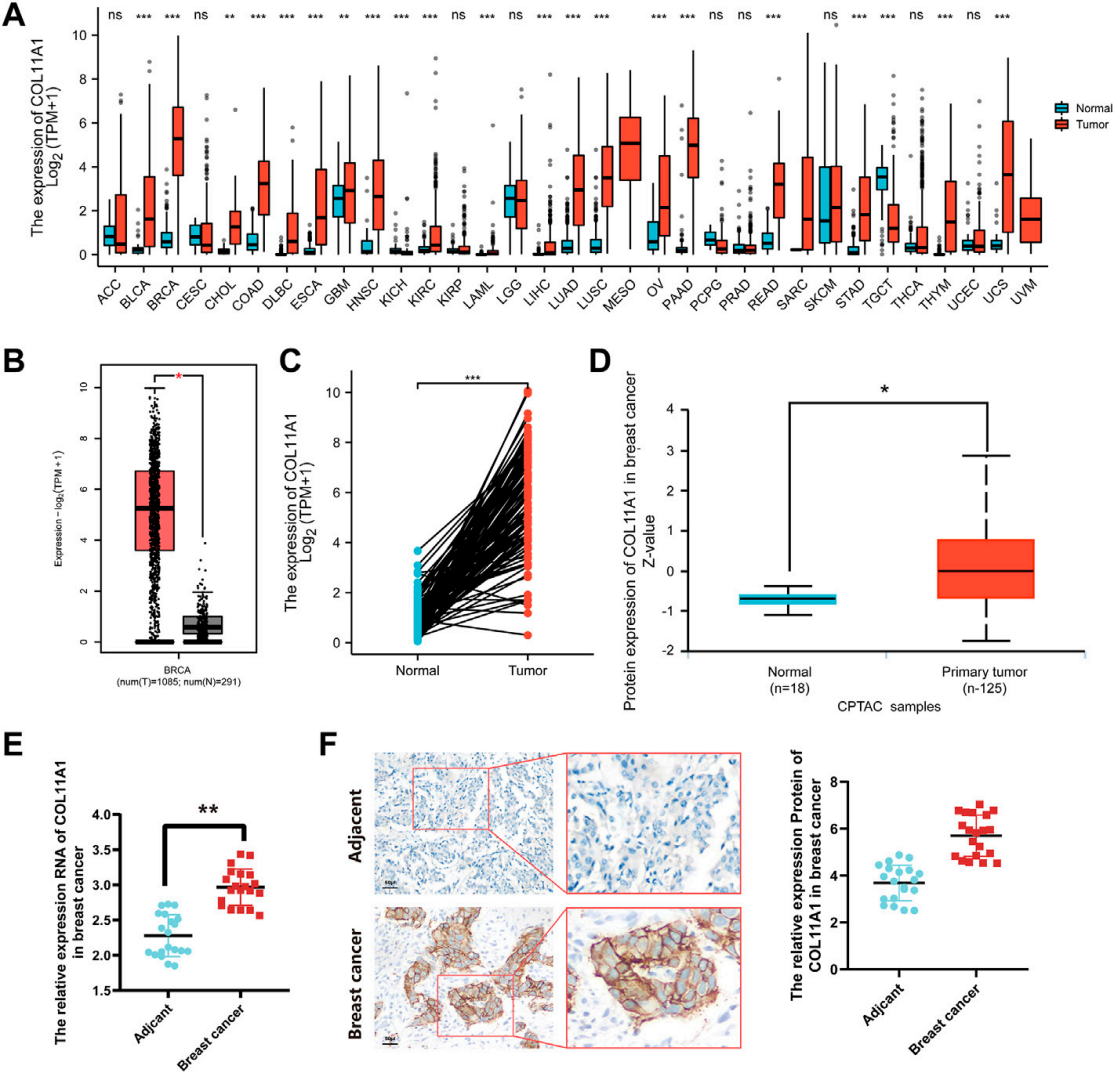
COL11A1 expression in breast cancer. **(A)** The expression of COL11A1 in different types of cancer was investigated using the TCGA database. **(B)** Increased expression of COL11A1 in breast cancer compared to normal tissues in the GEPIA2 database. **(C)** Statistical analysis of COL11A1 expression in TCGA database and 112 pairs of breast tissue and adjacent normal tissue. **(D)** The protein expression of COL11A1 in breast cancer was examined using the UALCAN database. *p < 0.05, ***p < 0.001. **(E)** RT-qPCR detection of COL11A1 transcript levels between breast cancer tissues and breast tissues. **(F)** Immunohistochemical staining was used to monitor COL11A1 protein levels between breast cancer tissues and breast tissues.

Gene expression arrays of 38 human tumors were downloaded from TCGA database, and the difference of COL11A1 expression between cancer tissues and corresponding adjacent tissues was analyzed by R language package DESeq2 and TIMER database. The results suggest that, compared with adjacent tissues, abnormal upregulation of COL11A1 has been observed in a variety of malignancies, including adrenal cortical carcinoma (ACC), bladder urothelial carcinoma (BLCA), invasive breast cancer (BRCA), cervical squamous carcinoma and adenocarcinoma (CESC), cholangiocarcinoma (CHOL), and colon adenocarcinoma (COAD) (Figure 1A, Supplementary Figure S1). In the Gene Expression Profile Interaction Analysis (GEPIA2) database and 111 pairs of cancer tissues and paired adjacent tissues isolated in the breast cancer gene expression array through the clinical sample Barcode officially provided by TCGA, we also consistently found that the expression of COL11A1 in BRCA tissues was higher than that in normal breast tissues (Figures 1B,C). To explore the difference in COL11A1 protein expression levels, we used UALCAN online tool to analyze invasive breast cancer and normal breast tissue samples from the CPTAC database. The results suggested that the protein level of COL11A1 in breast cancer tissues was significantly higher than that in normal breast tissues (Figure 1D). In order to further confirm the difference of COL11A1 expression between breast cancer and Adjacent tissues, 20 pairs of paired samples from breast cancer patients were detected, and the mRNA transcription and protein expression levels were detected by RT-qPCR and immunohistochemistry. The results showed that the levels of mRNA transcription and protein expression of COL11A1 in cancer tissues were significantly up-regulated compared with those in adjacent tissues (Figures 1E,F).

### Relationship between expression level of COL11A1 and clinical features of breast cancer patients

**FIGURE 2:**
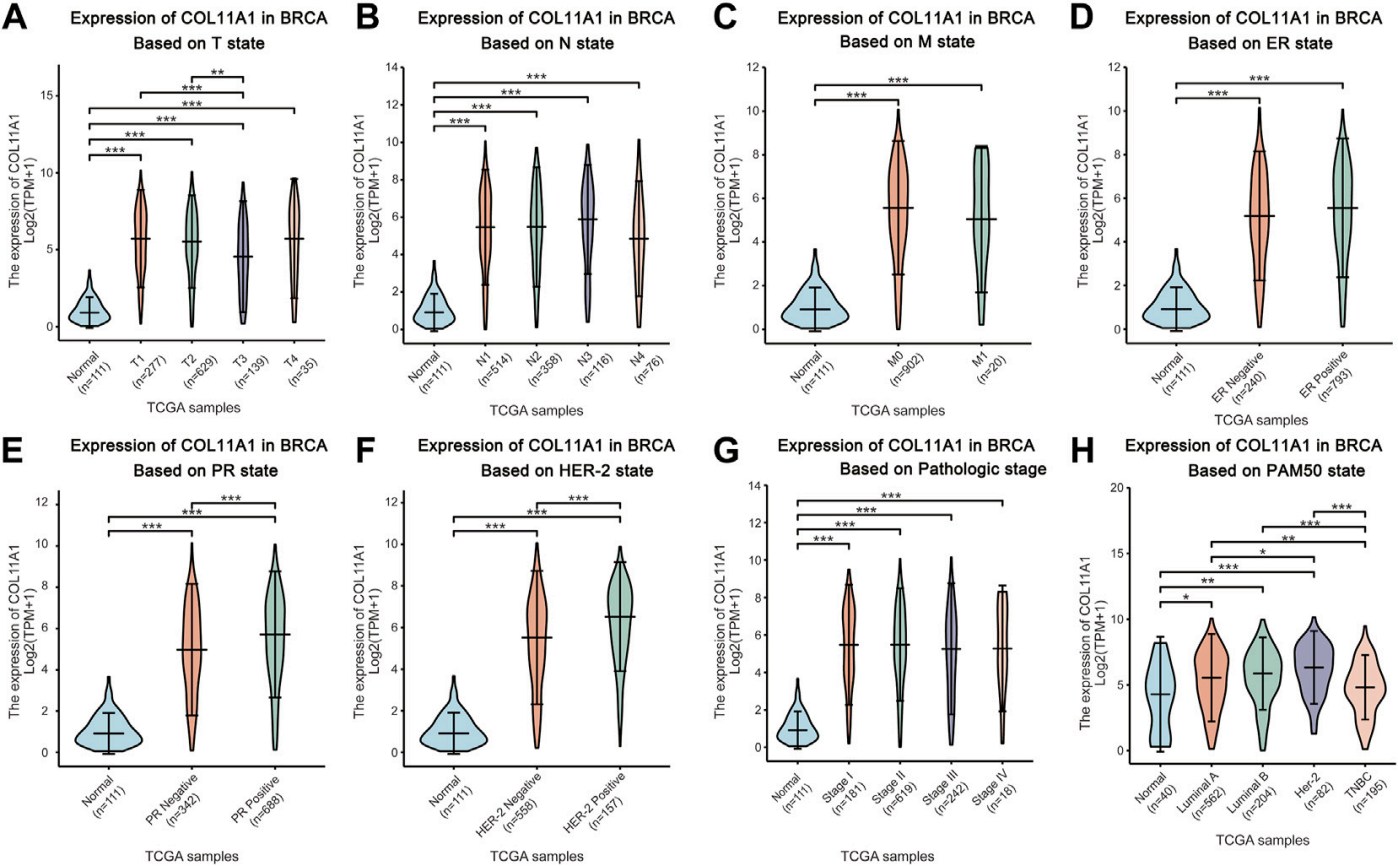
Box plots evaluating COL11A1 expression among different groups of patients based on clinical parameters using the TCGA database. Analysis is shown for T state **(A)**, N state **(B)**, M state **(C)**, ER state **(D)**, PR state **(E)**, Her-2 state **(F)**, breast cancer stage **(G)** and the PAM50 state **(H)**. *p < 0.05, **p < 0.01, ***p < 0.001.

The breast cancer samples with complete clinical information as well as the normal breast tissues were matched with the samples in the gene expression array through the TCGA official sample barcodes. We used one-way ANOVA to analyze the expression differences of COL11A1 among patients grouped according to different clinical parameters, and further used LSD method to compare the results of the differences among multiple groups. Our subsequent analysis showed that, after grouping according to tumor T status, N status, M status, ER status, PR status, HER-2 status, clinical stage, PAM50 status, histological type of breast cancer, and menopause status, compared with the control group, the expression level of COL11A1 in breast cancer tissues of each group was significantly increased (Figures 2A–H, Supplementary Figure S2). Among them, compared with PR and HER-2 negative breast cancer tissues, PR and HER-2 positive breast cancer tissues have higher expression levels of COL11A1 (Figures 2E,F). Compared with invasive lobular carcinoma, COL11A1 showed higher expression levels of COL11A1 in invasive ductal carcinoma tissues (Figure 2G). In addition to the elevated expression levels of COL11A1 in breast cancer tissues compared with normal breast tissues (There were also significant differences in COLL11A1 expression levels among breast cancer subtypes (Figure 2H).

### High expression of COL11A1 in patients with breast cancer is associated with adverse effects

**FIGURE 3:**
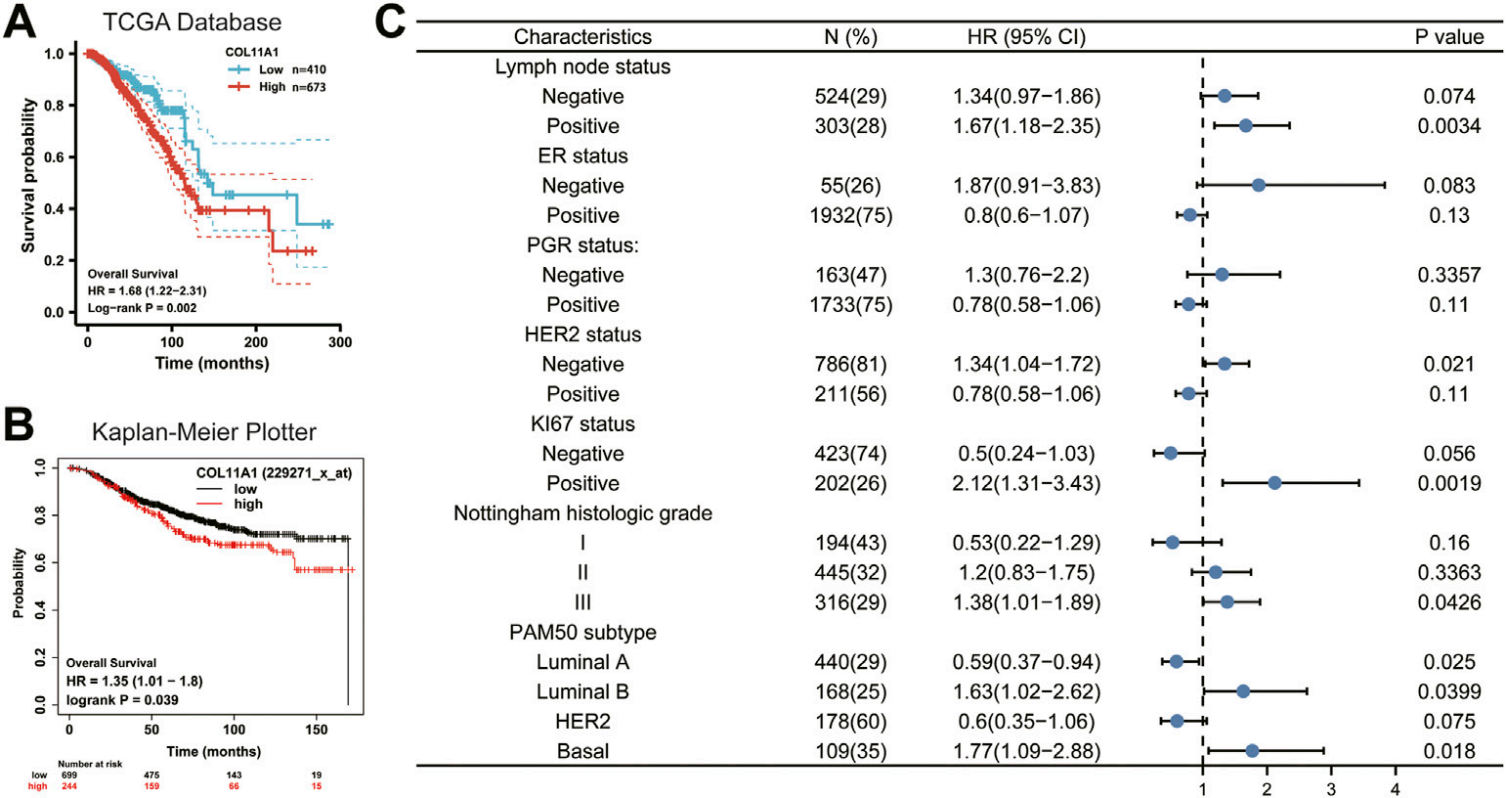
Survival curve evaluating the prognostic value of COL11A1. **(A)** Survival curve using the TCGA database is shown for OS. **(B)** Survival curve using the Kaplan-Meier plotter is shown for OS. **(C)** A forest plot shows the correlation between COL11A1 expression and clinicopathological parameters in BRCA patients.

Since the expression level of COL11A1 is closely related to the progression and metastasis of breast cancer, in order to further explore the predictive value of COL11A1 in the prognosis of breast cancer, we compared the overall survival (OS) of breast cancer patients with high expression of COL11A1 gene in the TCGA database and the Kaplan Meier Potter database. The analysis results of the two data consistently showed that high expression of COL11A1 was significantly associated with poor OS in breast cancer patients (Figures 3A,B). These results suggest that COL11A1 is closely related to the prognosis of breast cancer patients.

### Verification of prognostic value of COL11A1 based on different clinicopathological features

To better understand the prognostic value and possible mechanisms of COL11A1 expression in breast cancer, we explored the relationship between COL11A1 transcription level and clinical features using the Kaplan-Meier database. It is worth noting that among breast cancer patients with different lymph node status, only high expression of COL11A1 in patients with positive lymph node metastasis was significantly associated with poor OS of the patients (Figure 3C). Interestingly, the expression of ER and PGR did not affect the effect of high COL11A1 expression on OS in breast cancer patients. For those who with different HER-2 expression profiles, high expression of COL11A1 was associated with poor OS only in breast cancer patients without HER-2 overexpression (Figure 3C).

In patients with high expression levels of ki-67, COL11A1 was shown to be significantly associated with poor OS prognosis (Figure 3C). Furthermore, we found that in breast cancer patients with histological grades I, II, and III, COL11A1 was only associated with poor prognosis in patients with histological grade III (Figure 3C). More interestingly, among the 4 breast cancer subtypes classified according to the expression of PAM50, the overexpression of COL11A1 in Luminal A, Luminal B, and Basal breast cancers all showed a significant impact on the prognosis of patients. And the COL11A1 overexpression in Luminal B and Basal subtypes appeared as a risk factor, while in Luminal A, COL11A1 appeared as a protective factor (Figure 3C). The above results suggest that the expression of COL11A1 in breast cancer has a certain prognostic value.

### Identification of COL11A1-interacting genes and proteins and genetic alterations

**FIGURE 4:**
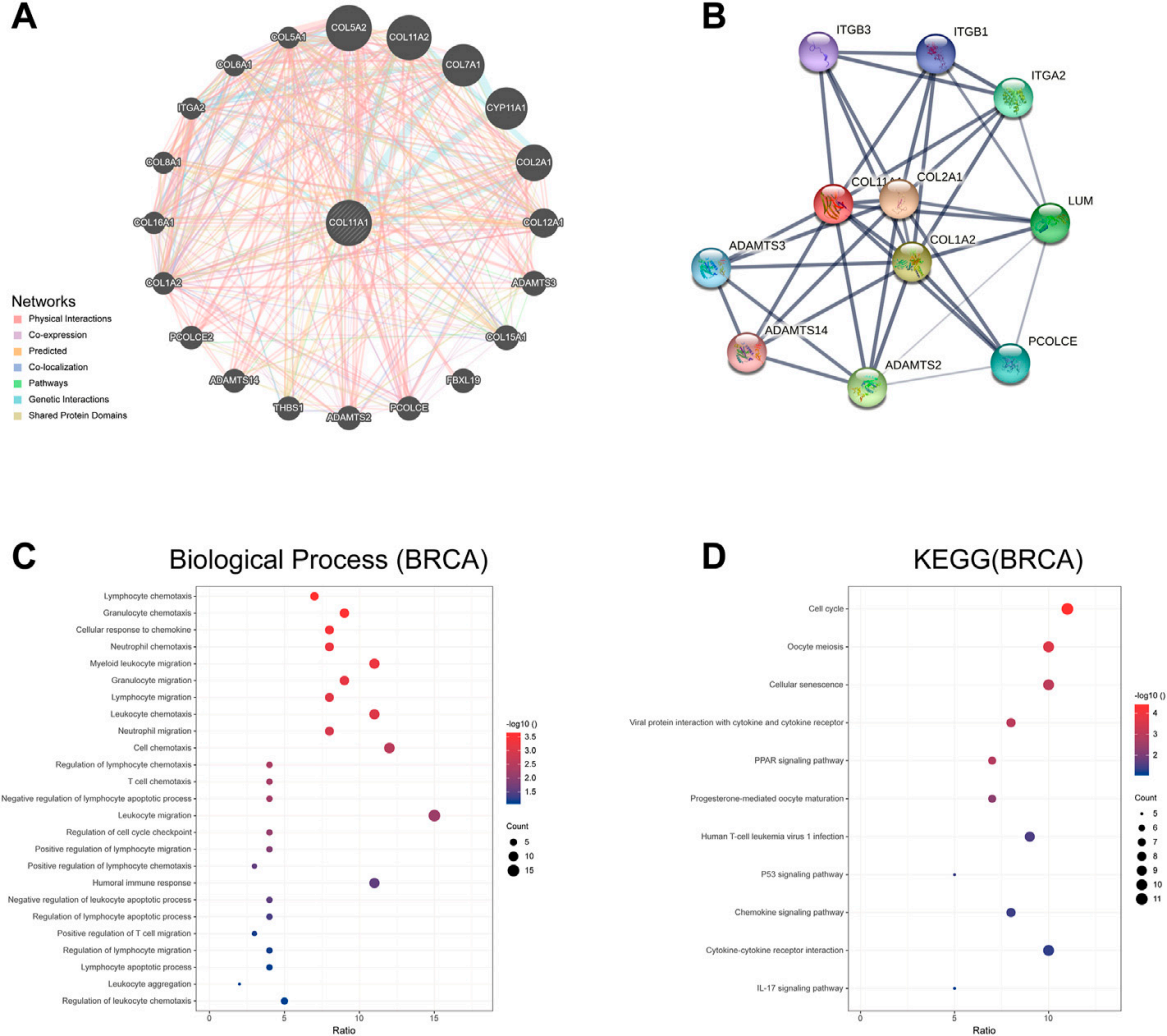
The genes and protein interaction network of COL11A1 **(A)** The gene-gene interaction network of COL11A1 was constructed using GeneMania. **(B)** The PPI network of COL11A1 was generated using STRING. **(C)** A bubble chart shows the GO analyses of 24 COL11A1 correlational genes. **(D)** A bubble chart shows the KEGG analyses of 24 COL11A1 correlational genes. *p < 0.05, **p < 0.01.

We used GeneMania to construct a gene-gene interaction network of COL11A1 and its neighboring genes. The results showed that the 20 genes most frequently associated with COL11A1 alterations included COL5A1,, COL7A1, CYP11A1, COL2A1 and ADAMTS3, etc (Figure 4A). A protein-protein interaction (PPI) network of COL11A1 was generated using the STRING database (Figure 4B). There are 38 edges and 11 nodes in total, including COL2A1, COL1A2, ADAMTS14 and ADAMTS3 and other proteins closely related to the changes of COL11A1 expression. We further conducted a further bioinformatics analysis on the genes closely related to COL11A1 (COL5A1, COL2A1, COL7A1), and the analysis results showed that these genes were differentially expressed in breast cancer, and all of them had important effects on the prognosis and tumor immune infiltration of breast cancer patients. Significant impact (Supplementary Figure S3). After sorting the genes in the two constructed networks, we used the R software package clusterProfiler package [version 3.14.3] for enrichment analysis. The enrichment results found that the biological functions (BP) of the genes in this group were mainly enriched in lymphocyte chemotaxis, granulocyte chemotaxis, cellular response to chemokine, neutrophil chemotaxis, T cell chemotaxis, humoral immune responsed and other immune response functions (Figure 4C). KEGG enrichment results show that this gene set is enriched in tumor-related signaling pathways like ECM-receptor interaction, Cell cycle, Viral protein interaction with cytokine and cytokine receptor, PPAR signaling pathway, PI3K-Akt signaling pathway, Human T-cell leukemia virus 1 infection, p53 signaling pathway, etc (Figure 4D).

### Gene ontology and kyoto encyclopedia of genes and genomes pathway analysis of COL11A1 and its co-expressed genes in TCGA breast cancer

**FIGURE 5:**
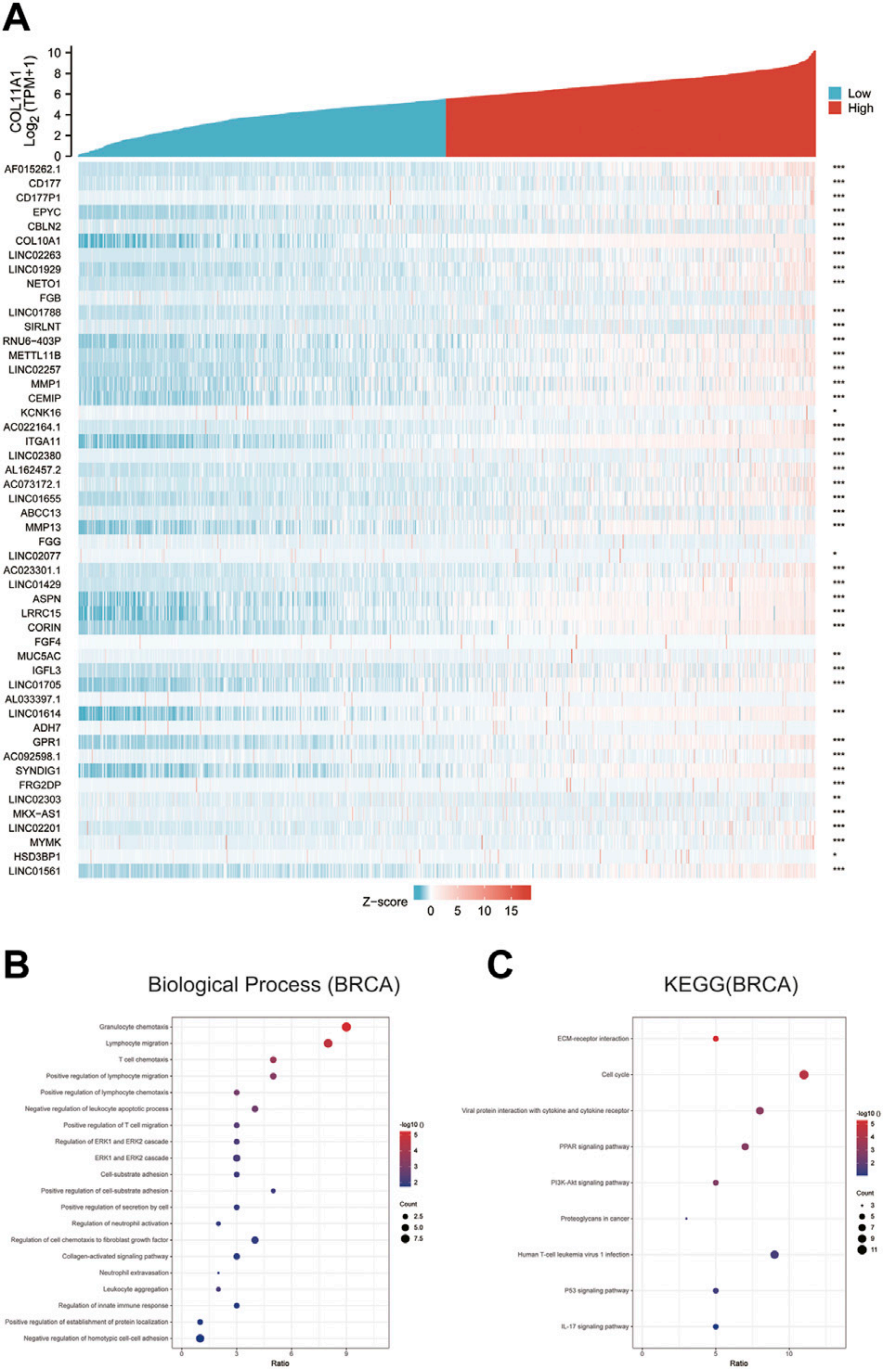
GO and KEGG enrichment analysis for COL11A1 correlational genes from TCGA database. **(A)** Heat maps showing the top 50 genes positively correlated with COL11A1 in BRCA. **(B)** The enrichment terms in BP categories in BRCA. **(C)** The KEGG enrichment pathways in BRCA.

The differentially expressed genes between breast cancer patients with high COL11A1 expression and those with low expression of COL11A1 were analyzed using the R software DEseq2 package in the TCGA database. The graph shows the top 50 genes positively or negatively associated with COL11A1 in breast cancer (Figure 5A). Then, GO and KEGG enrichment analysis was performed using the top 100 differentially expressed genes positively correlated to COL11A1. The figure lists the enrichment information of Biological Process (BP) and Molecular Function (MF). In terms of biological processes, COL11A1 and its related genes were significantly enriched in immune response-related processes, including lymphocyte chemotaxis, granulocyte chemotaxis, cellular response to chemokine, etc (Figure 5B). The KEGG of this part is also enriched in tumor and immune-related signaling pathways such as cell cycle, Oocyte meiosis, Cellular senescence, PPAR signaling pathway, Progesterone-mediated oocyte maturation, P53 signaling pathway, IL-17 signaling pathway, PI3K-Akt signaling pathway, etc (Figure 5C).

### Correlation of COL11A1 expression with various immune cell infiltration

**FIGURE 6:**
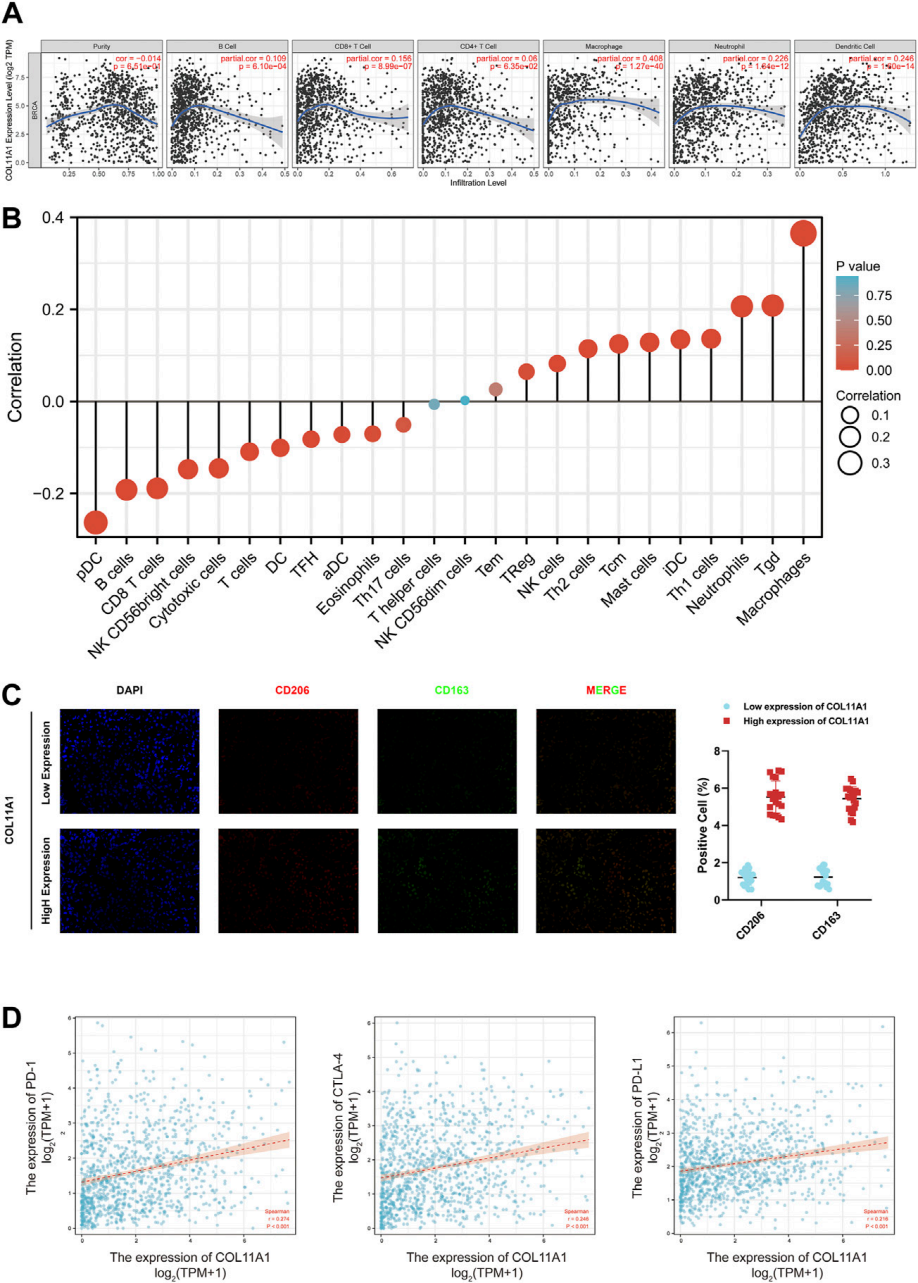
Correlation of COL11A1 expression with immune infiltration levels. **(A)** COL11A1 was significantly correlated with tumor purity and positively correlated with different immune cell infiltration using the TIMER database. **(B)** COL11A1 expression was significantly correlated with immune cell infiltration in breast cancer using the Lollipop chart. **(C)** Immunofluorescence staining labeled two biomarkers, CD206 and CD163, of macrophages in breast cancer tissues; **(D)** The correlation between COL11A1 expression and PD-1, PD-L1 and CTLA-4 in BRCA using TCGA database Scatter plot of correlations.

The correlation between the expression level of COL11A1 and 6 types of immune infiltration cells including B cells, CD4^+^ T cells, CD8^+^ T cells, neutrophils, macrophages and dendritic cells was analyzed in BRCA. We finished the correlation analysis in the TIMER database, which showed that the expression level of COL11A1 was positively correlated with the infiltration of B Cell, CD8^+^ T Cell, CD4^+^ Cell, macrophage, neutrophil and dendritic cell in BRCA (Figure 6A). It is worth noting that the expression of COL11A1 was particularly correlated with the infiltration of macrophages (Figure 6A). To further assess the impact of COL11A1 on the tumor microenvironment, we evaluated the correlation between COL11A1 and immune infiltration using the GSVA package [version 1.34.0]. It`s a remarkable fact that COL11A1 was negatively correlated with Treg cells, NK cells, Th2 cells, Tcm, Mast cells, iDC, TH1 cells, Neutrophils, Tgd, Macrophages, while it was positively related to pDC, B cells, CD8^+^ T cells, NK CD56bright cells, Cytotoxic cells, T cells, DC, TFH, Adc, Eosinophils (Figure 6B). Immunofluorescence detection of clinicopathological specimens also found that the immune infiltration level of M2-TAMs in breast cancer tissues with high COL11A1 expression was significantly higher than that in breast cancer tissues with low COL1A1 expression (Figure 6C).

### Correlation between COL11A1 expression and various immune markers

In order to dig deeper into the relationship between COL11A1 expression and immune infiltration, we used the TIMER database to verify the correlations among different immune signals in BRCA. The genes listed in Supplementary Table S1 are used to characterize immune cells, including B cells, T cells, CD8^+^ T cells, monocytes, tumor-associated macrophages, M1 macrophages, M2 macrophages, neutrophils, natural killers (NK) cells and dendritic cells. Tumor purity is an important factor affecting the analysis of immune infiltration in clinical cancer tissues, so after correction for tumor purity, different types of immune cells in invasive breast cancer were evaluated, and the expression of COL11A1 was significantly correlated with most immune markers (Supplementary Table S1).

To further understand the correlation between COL11A1 and breast cancer immune status, we analyzed the correlation of COL11A1 expression with immune infiltration of various functional T cell subtypes, and the results showed that COL11A1 expression was correlated with 29 markers, which were expressed in Th1, Th1-like cells, Th2 cells, Treg cells, resting Tregs, effector Treg cells, naive T cells, effector memory T cells, resistant memory T cells and exhausted T cells (Supplementary Table S2). After adjustment for tumor purity, it was significantly associated with 28 markers of BRCA (Supplementary Table S2). We further investigated the relationship between COL11A1 expression levels and classic T cell checkpoints, such as PD-1, PD-L1, CTLA-4. In breast cancer, the expression of COL11A1 was significantly correlated with the expression of PD-1, PD-L1 and CTLA-4 (Figure 6D). These results suggest that the expression of COL11A1 is closely related to tumor immune infiltration, and indicate that COL11A1 plays an important role in immune escape in the breast cancer tumor microenvironment.

### Prognostic analysis of COL11A1 expression based on immune cells in BRCA patients

**FIGURE 7:**
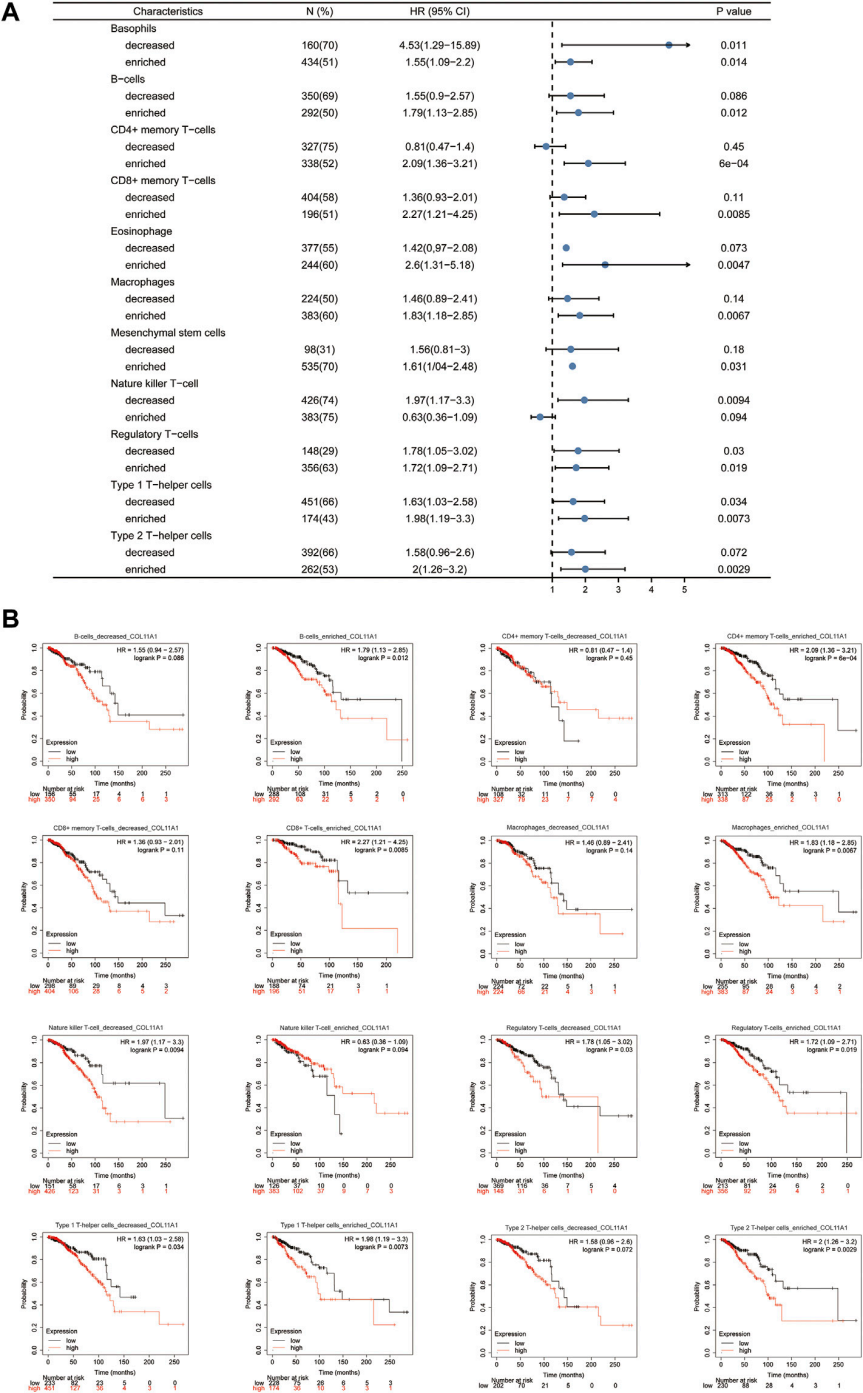
Kaplan-Meier survival curves according to high and low expression of COL11A1 in immune cell subgroups in breast cancer. **(A)** A forest plot shows the prognostic value of COL11A1 expression according to different immune cell subgroups in BRCA patients. **(B)** Correlations between COL11A1 expression and OS in different immune cell subgroups in BRCA patients were estimated by Kaplan-Meier plotter.

Given that the expression level of COL11A1 is closely related to the poor prognosis and immune infiltration of invasive breast cancer patients. To investigate in depth whether the effect of COL11A1 on the prognosis of invasive breast cancer is related to its involvement in regulating immune infiltration. We performed a prognostic analysis of subsets of immune cells within invasive breast cancer. As shown, decreased B cells and CD4^+^ memory T cell infiltration or increased macrophage and basophil infiltration associated with high COL11A1 expression suggest poor prognosis in breast cancer patients (Figure 7). These results suggest that the poor prognosis of breast cancer patients due to high COL11A1 expression may be closely related to the inhibition of intratumoral immune cell function by COL11A1.

## Discussion

According to the latest global cancer statistics, although the mortality rate of breast cancer still ranks fifth, its incidence rate has surpassed that of lung cancer and ranks first among human malignant tumors (Sung et al., 2021). High morbidity, mortality and disability rates have made breast cancer an unavoidable social problem for human beings (Trapani et al., 2022). Although we have made some progress in the diagnosis and treatment of early breast cancer through surgery, chemotherapy, radiotherapy, endocrine therapy combined with targeted therapy, the efficacy for advanced breast cancer is not optimistic (Han et al., 2022; Giordano et al., 2022; Meattini et al., 2022; Schneider et al., 2022). What is more worrying is that the latest data show that most of the patients diagnosed with breast cancer for the first time are in the advanced stage (Banham et al., 2019; Franzoi et al., 2019; Sabik et al., 2020; Trapani et al., 2022). As a systemic disease, breast cancer has been recognized by scholars around the world (Fisher et al., 1968; Fisher, 1969; Fisher, 1971), and surgical operation is the main mean in the treatment of breast cancer. However, surgery is only a local treatment, and the benefit of surgical treatment for breast cancer with distant metastasis is very limited. As endocrine therapy only plays a role in ER and PR-positive breast cancer patients (Burstein et al., 2016; Spring et al., 2016; Franzoi et al., 2021), the current rapid drug resistance of breast cancer to chemotherapy and endocrine therapy also greatly reduces the benefits of breast cancer patients (Nakao et al., 2018; Garcia-Martinez et al., 2021; Lloyd et al., 2022). Therefore, immunotherapy may be a potentially effective systemic therapy for breast cancer. However, immunotherapy for breast cancer has been carried out for several decades, there are still few therapeutic strategies implemented in clinical practice, and even fewer strategies that can produce good therapeutic effects (Zhu et al., 2021b; Loi et al., 2021; Xu et al., 2021). In addition, breast cancer is considered as a highly heterogeneous malignant tumor, and the differences between individualized treatment of breast cancer are difficulties in the diagnosis and treatment progress (Liang et al., 2020). Relevant studies indicate that finding new therapeutic targets for breast cancer may be a correct strategy to address the heterogeneity of breast cancer (Appierto et al., 2017). Therefore, it is of great significance to explore the mechanism of occurrence and development of breast cancer and to search for biomarkers related to breast cancer, so as to develop strategies for diagnosis, treatment and prognosis evaluation of breast cancer.

In this study, we used bioinformatics analysis of TCGA, UALCAN, CPTAC and GEPIA2 databases to find that COL11A1 is abnormally upregulated in a variety of human tumors (Figure 1), which is consistent with the results of many previous studies (Wu et al., 2021a; Lee et al., 2021; Ma et al., 2021). When we focused on breast cancer, we first compared the differences of COL11A1 in independent samples or paired samples in online tumor databases, and then we collected clinicopathological specimens and cultured breast cancer cell lines by immunohistochemical staining and Western Blot experiments, respectively. The protein expression level of COL11A1 in them was detected. During the experiment, we found that the two conclusions were consistent that the expression level of COL11A1 in the primary breast cancer was significantly lower than that in normal breast tissue or paracancerous tissue (Figure 1 & Supplementary Figure S4), whether in independent samples or paired samples. Methylation is an important modification of DNA in eukaryotes, and it is involved in the expression and regulation of most genes in eukaryotes. The effect of basalization differences on patient prognosis. Interestingly, we observed that patients with higher COL11A1 methylation had a worse prognosis (Supplementary Figure S4), which also suggested that methylation may play a role in the expression of COL11A1. The effect of basalization differences on patient prognosis. Subsequently, we studied the relationships between differential expression of COL11A1 and the corresponding T status, N status, M status, clinical stage, ER status, PR status, HER-2 status, PAM50 status, histological types and menopause status in breast cancer patients. Compared with the control group, there were significant differences in breast cancer of the above different states (Figure 2). Further overall survival analysis of COL11A1-related breast cancer patients showed that breast cancer patients with high COLL1A1 expression had worse overall survival than those with low COL11A1 expression (Figure 3). In order to clarify the impact of COL11A1 on the clinical progression of breast cancer, we conducted subgroup analysis based on lymph node status, estrogen, progesterone, histological grade and PAM50 status. And the relevant results (Figure 3) suggested that the different expression of COL11A1 has a higher value in evaluating the adverse prognosis of breast cancer patients with lymph node metastasis and increased histological grade. Interestingly, the prognostic value of COL11A1 in patients with different molecular subtypes of breast cancer also showed significant differences. These results indicate that COL11A1 is an important regulatory factor in the occurrence and development of breast cancer, and even COL11A1 may be an independent prognostic biomarker of breast cancer, which has the value as a potential target for diagnosis, treatment and prognostic evaluation of breast cancer.

COL11A1, as an important member of the collagen family, has a molecular weight of about 181065Da and is composed of 1806 amino acids (see Support Material 1 for detailed THREE-DIMENSIONAL structure). The level of COL11A1 is low when in normal physiological station, but as an important component of fibrin XI, the diameter of collagen fibers in various soft tissues (especially chondrogenesis) is regulated though COL11A1 (Sun et al., 2020). Pathologically, a variety of developmental malformations and structural abnormalities of organs are caused by loss of COL11A1, including type II Stickler syndrome and Marshall syndrome. Existing studies have shown that abnormal upregulation of COL11A1 may be associated with multiple tumor types, which was also confirmed by the pan-cancer analysis in this study. In ovarian cancer tissues, miR-335 inhibits the expression of COL11A1 and thus weakens the invasion ability of ovarian cancer (Wu et al., 2021a). COL11A1 induces the translocation of p65 into the nucleus via activation-mediated extracellular signal-regulated kinase (ERK) and enhances its binding to the insulin-like growth factor binding protein 2 (IGFBP2) promoter, ultimately inducing TGF-β3 activation to activate tumor-related fibroblasts and promote tumor progression (Wu et al., 2021b). Akt inhibitor SC66 reverses the cisplatin resistance in ovarian cancer by inhibiting COL11A1 (Wu et al., 2019). In lung cancer, mir-144–3p can inhibit the proliferation, invasion and migration of lung adenocarcinoma by targeting COL11A1 to down-regulate its expression level (Sun et al., 2021; Tu et al., 2021). Similar to its role in ovarian cancer, in addition to the regulation of invasion, migration and proliferation of tumor cells in NSCLC, COL11A1 induces the cisplatin resistance of tumor cells through overexpression and activating Smad signaling pathway (Shen et al., 2016). However, there are few studies on COL11A1 in breast cancer. Although previous studies have pointed out that abnormal expression of COL11A1 can change the biological characteristics of breast cancer cells (Gu et al., 2019), there is no report on the involvement of COL11A1 in the occurrence and development of breast cancer and its corresponding underlying mechanisms. Nevertheless, according to previous preliminary findings, the expression level of COL11A1 in breast cancer tissues was abnormally up-regulated, and high level of COL11A1 suggested poor prognosis of breast cancer patients (Figure 3). We believe that COL11A1 may play an important role in the occurrence and development of breast cancer. Therefore, COL11A1 may be a potential biomarker related to diagnosis, treatment and prognosis assessment of breast cancer.

To further confirm the role of COL11A1 in the occurrence and development of breast cancer, we constructed a gene network interacting with COL11A1 in GeneMania database, and listed the top 20 genes closely related to COL11A1 (Figure 4). In the STRING database, the protein interaction network is constructed. The gene sets involved in the two databases were combined, then the biological process (BP), molecular function (MF) enrichment analysis of the union were performed, as well as the related signaling pathways (MF was shown in the supporting materials). Enrichment analysis results suggest that the gene sets are enriched in the biological process of Lymphocyte chemotaxis, Granulocyte chemotaxis, Cellular Response chemokine, neutrophil chemotaxis, myeloid leukocyte migration, humoral immune response and so on. (Figure 4); The results of signaling pathway enrichment analysis suggest that this gene set is enriched in Cell cycle, Oocyte meiosis, Cellular senescence, Viral protein interaction with cytokine and cytokine receptor, PPAR signaling pathway, Human T cell leukemia virus I infection, P53 signaling Pathway, Chemokine Signaling Pathway, etc. In order to further search for COL11A1-related differentially expressed genes, we compared COL11A1-related differentially expressed genes in TCGA data and used DEseq2 R software package, and listed the top 50 genes positively correlated with COL11A1 expression level. In order to explore the biological functions of COL11A1 co-expressed gene groups, the top 100 gene groups positively correlated with COL11A1 were selected for GO and KEGG enrichment analysis. Enrichment analysis showed that the biological processes of the 100 positively correlated differentially expressed genes were mainly enriched in Granulocyte chemotaxis, lymphocyte migration, T cell chemotaxis, positive regulation of lymphocyte migration, positive regulation of lymphocyte chemotaxis, negative regulation of leukocyte apoptotic process, positive regulation of T cell migration, Regulation of ERK1 and ERK2 cascade and other immune processes (Figure 5). Key words related to KEGG enriched signaling pathways mainly include ECM-receptor interaction, Cell cycle, Viral protein interaction with cytokine and cytokine receptor, PPAR signaling pathway, PI3K-Akt signaling pathway, P53 signaling Pathway, etc. It is well known that PARP and PI3K are signaling pathways closely related to the invasion and migration of breast cancer. The results of the enrichment analysis of the signaling pathways in this study suggest that COLL1A1 may be closely related to the invasion and migration of breast cancer. According to existing studies, COL11A1 can serve as a downstream target gene of multiple non-coding RNAs. Among them, Gu’s study showed that up-regulation of the tumor suppressor gene miR-139–5p can significantly inhibit the expression level of COL11A1, thereby inhibiting proliferation and promoting apoptosis (Gu et al., 2019). In addition, Liu inhibited the progression of estrogen receptor-positive breast cancer by constructing MiR-4458-loaded gelatin nanospheres targeting COL11A1 to block the DDR2/SRC signaling pathway (Liu et al., 2022). Therefore, it is not difficult to find that COL11A1 is involved in the occurrence and development of various breast cancers including invasion and metastasis, but whether it is involved in DNA repair, tumor microenvironment immunity and other biological processes in breast cancer progression has not been reported. Interestingly, existing research reports show that COLL1A1 can also be regulated by coding genes in addition to non-coding RNA regulation. Wang reported that CDX2 overexpression could alleviate breast cancer progression by upregulating microRNA let-7b and inhibiting COL11A1 expression (Wang et al., 2020a). Accordingly, we believe that COL11A1 under the regulation of non-coding genes and coding genes plays an important role in the occurrence and development of breast cancer. The biological process of the molecular network related to the gene-protein interaction network constructed by the comprehensive GeneMania and STRING databases, the biological enrichment process of the differentially expressed gene set and the enrichment results of the signaling pathway and the existing literature showed that the abnormal expression of COL11A1 is closely related to the invasion and metastasis of breast cancer. Although no studies have reported the relationship between COL11A1 and breast cancer tumor immunity, two gene groups closely related to COL11A1 are significantly enriched in immune-related biological processes. Therefore, we speculate that COL11A1 may be involved in the biological process of breast cancer tumor immunity, but this conclusion still needs further biological experiments to prove.

In order to confirm the role of COL11A1 in the tumor immune process of breast cancer, we introduced the TIMER database, and explored the correlation between COL11A1 expression level and tumor immune cell infiltration in the TIMER data. It is not hard to find that COL11A1 is closely related to the infiltration of various tumor immune cells including B Cell, CD8 + T cell, CD4 + T cell, macrophage and neutrophil and dendritic cell. In particular, the expression level of COL11A1 was positively correlated with the infiltration degree of macrophages. In order to clarify the immune cell subsets mainly affected by COL11A1, we detected the expression of COL11A1 and biomarkers related to each immune cell subset, and performed correlation analysis. We found that COL11A1 was significantly associated with the infiltration level of macrophages, especially type 2 tumor-associated macrophages. According to existing literature, M2-TAMS often plays a disgraceful role in tumor immunity. For example, in prostate cancer, high M2-TAMs infiltrating subtypes have the worst prognosis and the weakest intratumoral immunity among the three subtypes of prostate cancer, indicating a low tumor response rate against PD-1 therapy (Jiawei et al., 2021). In colorectal cancer, M2-TAMs induced by Wnt5a-mediated CaKMII-ERK1/2-STAT3 pathway-mediated IL-10 secretion can significantly promote the growth and metastasis of intestinal cancer cells (Liu et al., 2020). In addition, in this study, we observed significant correlation between COL11A1 expression levels and a variety of immune cell markers in TCGA data (Supplementary Table S1). In this study, we noted that T cell-related markers, the main force in tumor immunity, were negatively correlated with COL11A1 expression. In order to identify the T cell subsets associated with COL11A1, we further compared the correlation between the signature biomarkers of each T cell subsets and COL11A1 expression (Supplementary Table S2). It is not difficult to find that biomarkers such as Effector T-cell, Naïve T -cell, Effector Memory T-cell and Resident Memory T-cell related to tumor immunity in T cell subsets are negatively correlated with COL11A1 expression while the infiltration level of Treg and Resting Treg (Zhang et al., 2021; Malla et al., 2022) that have been previously confirmed by a large number of literatures to have tumor immunosuppressive effect and participate in tumor progression, were positively correlated with COL11A1 expression. In addition, we unexpectedly found a positive correlation between COL11A1 and the expression of PD-1, PD-L1 and CTLA-4 (Figure 6D). Accordingly, we propose that COL11A1 may be a very promising new immunotherapy-related target for breast cancer. However, the specific molecular mechanism of how COL11A1 participates in immune cell regulation in tumor microenvironment still needs to be further explored and verified by biological experiments. More importantly, we further found that COL11A1 was associated with the prognosis of breast cancer patients, possibly due to its involvement in the regulation of tumor immunosuppression (Figure 7).

In summary, this study has improved our understanding of the relationship between COL11A1 and breast cancer, but this study is not absolutely perfect. First of all, as breast cancer is a highly heterogeneous tumor, the pathological process of different molecular subtypes of breast cancer is significantly different, but we didn`t discuss each molecular subtype respectively in this study. Secondly, as mentioned above, this study did reveal the correlation between COL11A1 and breast cancer tumor immunity and confirmed the main immune cell subsets regulated by COL11A1, but we did not elaborate on its specific regulatory mechanism. Thirdly, most of the analysis in this study were based on the transcription level of COL11A1mRNA, and there was a lack of verification of the corresponding protein level. Overall, our results suggest that COL11A1 may be a potential new prognostic biomarker for breast cancer. In addition, we explored potential evidence that COL11A1 regulates immune cell infiltration in breast cancer TME. Therefore, these findings not only add to our current understanding of the role of COL11A1 in breast cancer, but also confirm its potential value in breast cancer prognosis and immunotherapy.

## Data Availability

The datasets presented in this study can be found in online repositories. The names of the repository/repositories and accession number(s) can be found in the article/Supplementary Material.
