# Supplementary figures and images for: COL11A1 serves as a biomarker for poor prognosis and correlates with immune infiltration in breast cancer
Source: Front Genet. 2022 Sep 9;13:935860. doi: 10.3389/fgene.2022.935860 (PMC9500398; doi:10.3389/fgene.2022.935860)

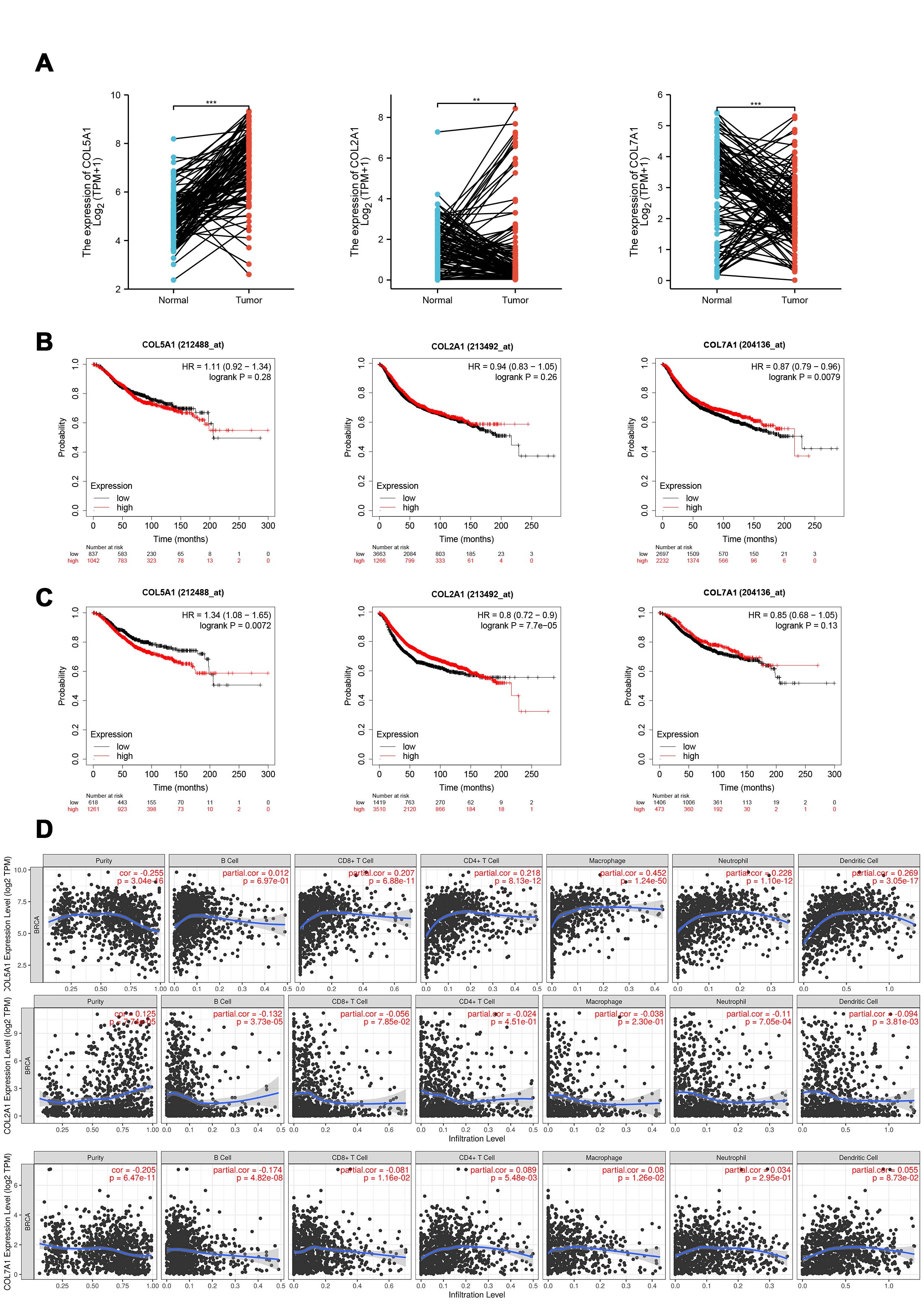

Supplement: Supplementary file 1 [file Image3.JPEG]

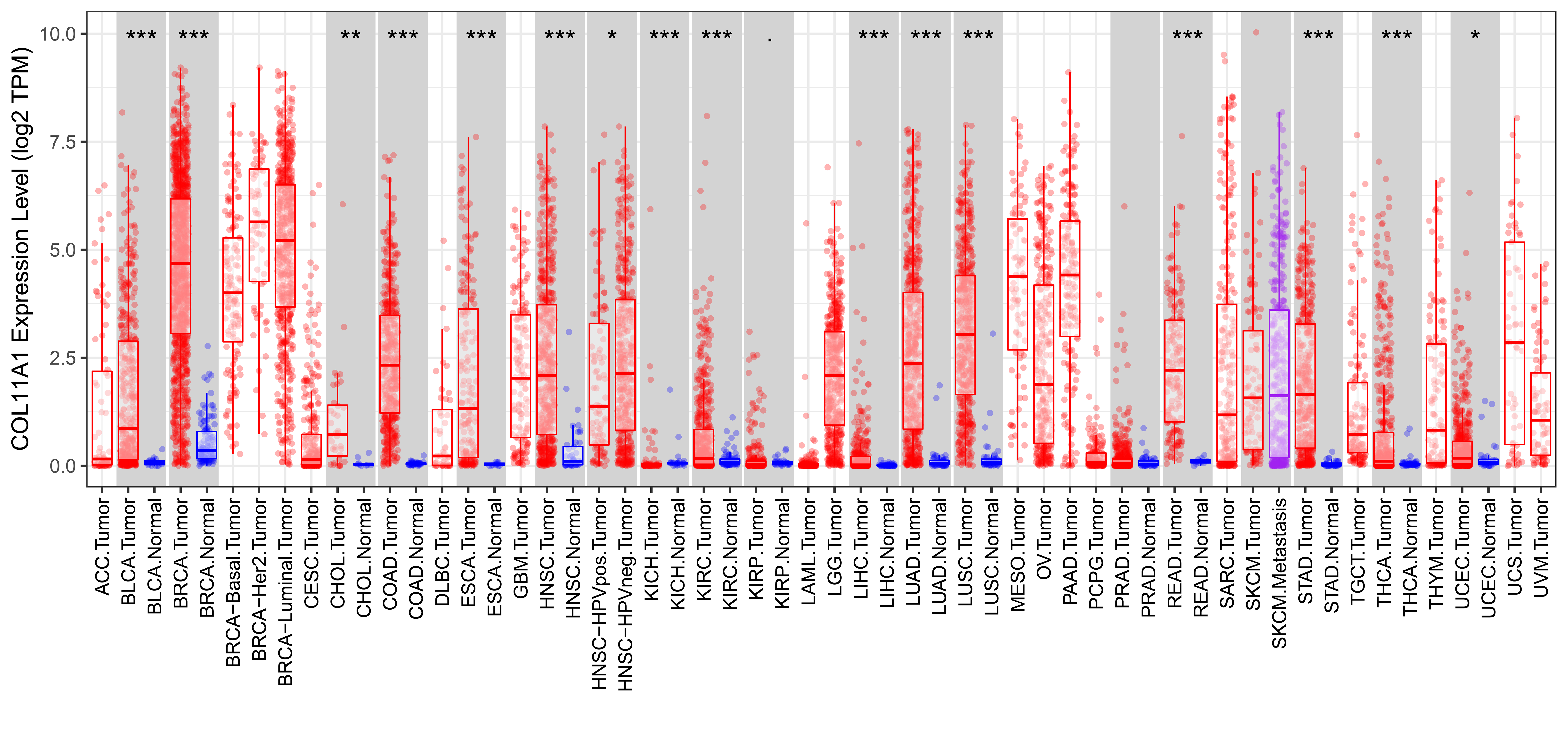

Supplement: Supplementary file 3 [file Image1.JPEG]

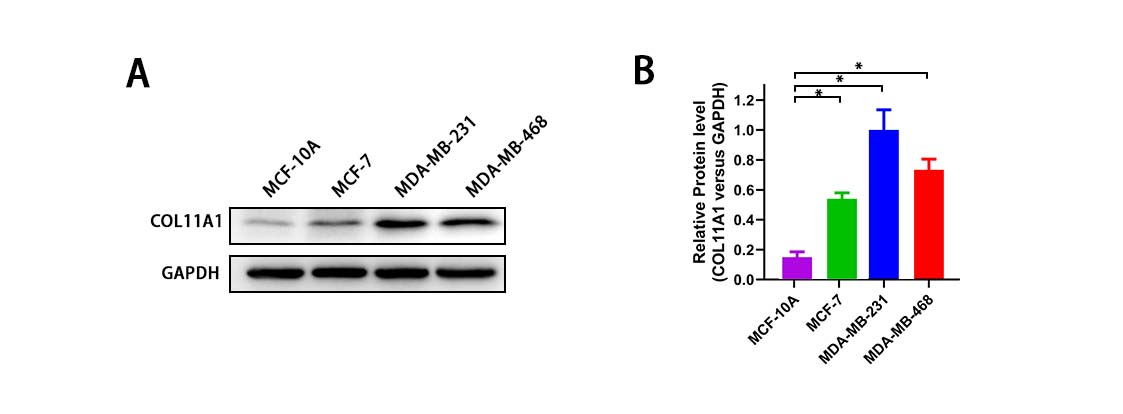

Supplement: Supplementary file 4 [file Image4.JPEG]

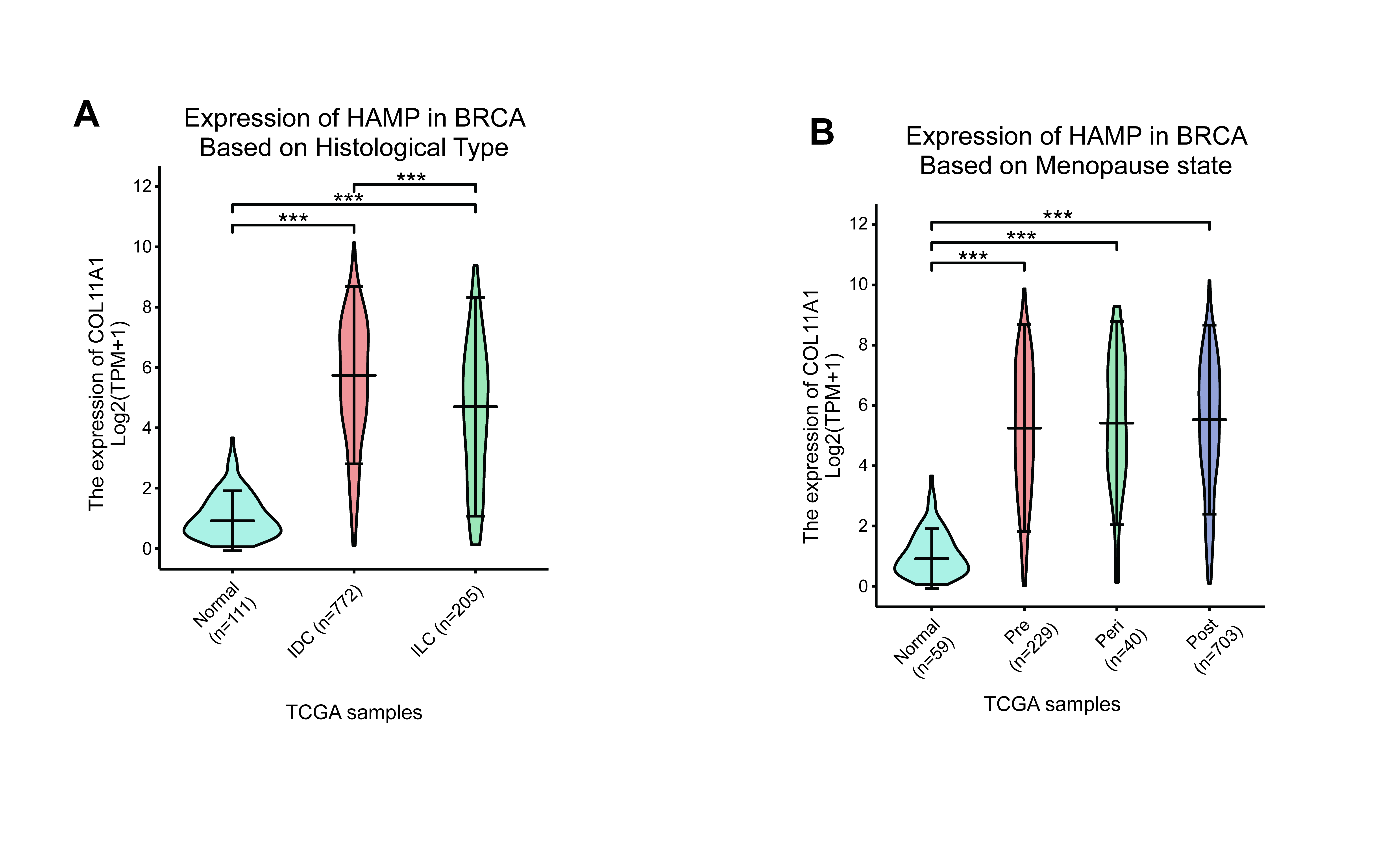

Supplement: Supplementary file 5 [file Image2.JPEG]

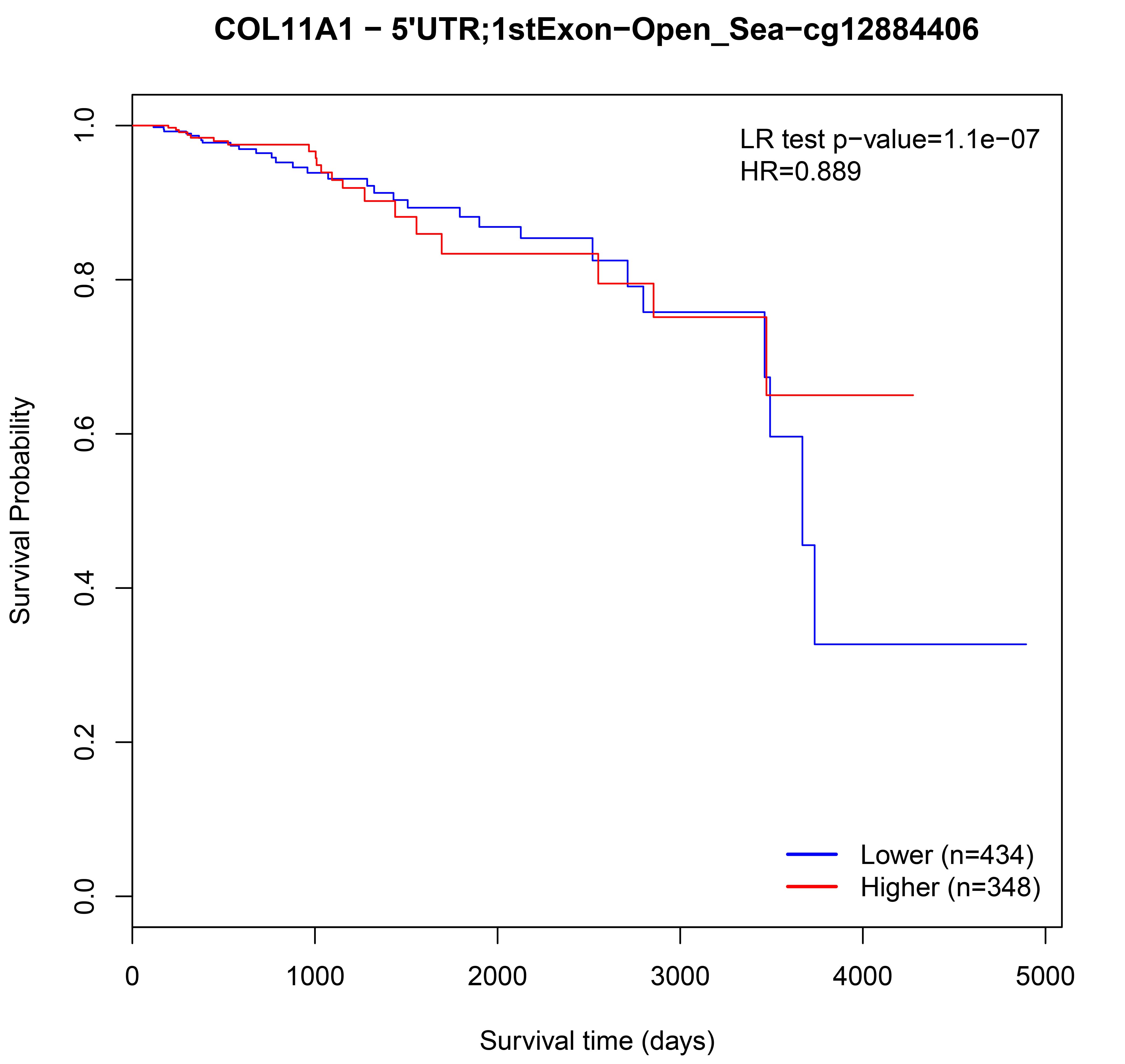

Supplement: Supplementary file 6 [file Image5.JPEG]
