# Supplementary material for: COL11A1 serves as a biomarker for poor prognosis and correlates with immune infiltration in breast cancer
Source: Front Genet. 2022 Sep 9;13:935860. doi: 10.3389/fgene.2022.935860 (PMC9500398; doi:10.3389/fgene.2022.935860)
Supplement: Supplementary file 2 [file Table1.DOCX]

Supplementary data

List of primers: used in RT-qPCR

| Name | Sequence 5’ – 3’ |
| --- | --- |
| COL11QA1 – F | GTCTGTTGGTCCAGTTGGTC |
| COL11QA1 – F | TTCTCTCCTCTTTCTCCTTTTGG |
| GAPDH - F | AGGTCGGAGTCAACGGATTT |
| GAPDH - R | TGACGGTGCCATGGAATTTG |
