# Supplementary material for: COL11A1 serves as a biomarker for poor prognosis and correlates with immune infiltration in breast cancer
Source: Front Genet. 2022 Sep 9;13:935860. doi: 10.3389/fgene.2022.935860 (PMC9500398; doi:10.3389/fgene.2022.935860)
Supplement: Supplementary file 7 [file Table2.DOCX]

| **Table 1. The correlation analysis between COL11A1 and Markers of immune Cells in TIMER.** | | | | | |
| --- | --- | --- | --- | --- | --- |
| **Description** | **Genn markers** | **BRCA** | | | |
|  |  | **None** | | **Purty** | |
|  |  | **Cor** | **p** | **Cor** | **p** |
| B cell | CD19 | -0.133 | *** | -0.156 | *** |
|  | CD79A | -0.118 | *** | -0.14 | *** |
| T cell (general) | CD3D | -0.087 | ** | -0.107 | *** |
|  | CD3E | -0.073 | * | -0.093 | ** |
|  | CD2 | -0.027 | 0.367 | -0.036 | 0.256 |
| CD8+ T cell | CD8A | -0.109 | *** | -0.13 | *** |
|  | CD8B | -0.152 | *** | -0.175 | *** |
| Monocyte | CD86 | 0.252 | *** | 0.274 | *** |
|  | CSF1R | 0.189 | *** | 0.206 | *** |
| TAM | CCL2 | 0.078 | *** | 0.076 | ** |
|  | CD68 | 0.326 | *** | 0.349 | *** |
|  | IL10 | 0.184 | *** | 0.191 | *** |
| M1 | IRF5 | 0.015 | 0.621 | 0.012 | 0.709 |
|  | PTGS2 | 0.029 | 0.329 | -0.033 | 0.292 |
|  | NOS2 | 0.284 | *** | 0.284 | *** |
| M2 | CD163 | 0.294 | *** | 0.305 | *** |
|  | VSIG4 | 0.311 | *** | 0.318 | *** |
|  | MS4A4A | 0.249 | *** | 0.26 | *** |
| Neutrophils | CEACAM8 | -0.067 | * | -0.043 | 0.171 |
|  | ITGAM | 0.311 | *** | 0.321 | *** |
|  | CCR7 | -0.172 | *** | -0.207 | *** |
| Natural killer cell | KIR2DL1 | -0.052 | 0.0829 | -0.069 | * |
|  | KIR2DL3 | 0.002 | 0.954 | 0.002 | 0.951 |
|  | KIR2DL4 | -0.104 | *** | -0.105 | *** |
|  | KIR3DL1 | -0.024 | 0.431 | -0.03 | 0.349 |
|  | KIR3DL2 | -0.079 | ** | -0.076 | * |
|  | KIR3DL3 | -0.046 | 0.127 | -0.055 | 0.0839 |
|  | KIR2DS4 | -0.011 | 0.712 | -0.008 | 0.8 |
| Dendritic cell | HLA-DPB1 | 0.039 | 0.198 | 0.038 | 0.228 |
|  | HLA-DQB1 | 0.074 | * | -0.059 | 0.0622 |
|  | HLA-DRA | 0.16 | *** | 0.181 | *** |
|  | HLA-DPA1 | 0.123 | *** | 0.133 | *** |
|  | CD1C | -0.137 | *** | -0.159 | *** |
|  | NRP1 | 0.391 | *** | 0.403 | *** |
|  | ITGAX | 0.272 | *** | 0.301 | *** |

| **Table 2. Correlation analysis between COL11A1 and gene markers of different types of T cells in TIMER** | | | | | |
| --- | --- | --- | --- | --- | --- |
| **Description** | **Gene markers** | **BRCA** | | | |
|  |  | **None** | | **Purity** | |
|  |  | **Cor** | **p** | **Cor** | **P** |
| Th1 | TBX21 | -0.119 | *** | -0.145 | *** |
|  | STAT4 | -0.006 | 0.855 | -0.01 | 0.761 |
|  | STAT1 | 0.148 | *** | 0.149 | *** |
|  | TNF | 0.062 | * | -0.062 | * |
|  | IFNG | -0.034 | 0.11 | -0.042 | 0,181 |
| Th1-like | HAVCR2 | 0.361 | *** | 0.385 | *** |
|  | IFNG | -0.034 | 0.11 | -0.042 | 0.181 |
|  | CXCR3 | -0.039 | 0.192 | -0.051 | 0.108 |
|  | BHLHE40 | 0.137 | *** | 0.14 | *** |
|  | CD4 | 0.175 | *** | 0.196 | *** |
| Th2 | STAT6 | -0.025 | 0.416 | -0.033 | 0.302 |
|  | STAT5A | -0.033 | 0.272 | -0.039 | 0.223 |
| Treg | FOXP3 | 0.152 | *** | 0.165 | *** |
|  | CCR8 | 0.264 | *** | 0.274 | *** |
|  | TGFB1 | 0.28 | *** | 0.287 | *** |
| Resting Treg | FOXP3 | 0.152 | *** | 0.165 | *** |
|  | IL2RA | 0.132 | *** | 0.143 | *** |
| Effector Treg T-cell | FOXP3 | 0.152 | *** | 0.165 | *** |
|  | CCR8 | 0.264 | *** | 0.274 | *** |
|  | TNFRSF9 | 0.245 | *** | 0.267 | *** |
| Effector T-cell | CX3CR1 | 0.136 | *** | 0.133 | *** |
|  | FGFBP2 | -0.2 | *** | -0.22 | *** |
|  | FCGR3A | 0.443 | *** | 0.456 | *** |
| Naïve T-cell | CCR7 | -0,172 | *** | -0.207 | *** |
|  | SELL | -0.104 | *** | -0.122 | *** |
| Effector Memory T-cell | DUSP4 | 0.049 | 0.106 | 0.053 | 0.0928 |
|  | GZMK | -0.09 | ** | -0.111 | *** |
|  | GZMA | -0.065 | * | -0.08 | * |
| Resident memory T-cell | CD69 | -0.052 | 0.0832 | -0.057 | 0.0715 |
|  | CXCR6 | -0.013 | 0.667 | -0.02 | 0.534 |
|  | MYADM | 0.242 | *** | 0.231 | *** |
|  | CCR7 | -0.172 | *** | -0.207 | *** |
| General memory T-cell | SELL | -0.104 | *** | -0.122 | *** |
|  | IL7R | 0.117 | *** | 0.136 | *** |
| Exhausted T-cell | HAVCR2 | 0.361 | *** | 0.385 | *** |
|  | LAG3 | -0.087 | ** | -0.096 | ** |
|  | CXCL13 | -0.078 | ** | -0.089 | ** |
|  | LAYN | 0.395 | *** | 0.411 | *** |
